# Revisiting the phylogeny and taxonomy of the Pithecellobium clade (Leguminosae, Caesalpinioideae) with new generic circumscriptions

**DOI:** 10.3897/phytokeys.205.82728

**Published:** 2022-08-22

**Authors:** Iván Tamayo-Cen, Benjamin M. Torke, José Enrique López Contreras, German Carnevali Fernández-Concha, Ivón Ramírez Morillo, Lilia Lorena Can Itza, Rodrigo Duno de Stefano

**Affiliations:** 1 Herbarium CICY, Centro de Investigación Científica de Yucatán, A. C. (CICY), Calle 43 No. 130, Colonia Chuburná de Hidalgo, CP 97200, Mérida, Yucatán, Mexico Centro de Investigación Científica de Yucatán Mérida Mexico; 2 Institute of Systematic Botany, New York Botanical Garden, 2900 Southern Boulevard, Bronx, New York, 10458-5126 USA New York Botanical Garden New York United States of America; 3 Centro de Investigación de Ciencias Ambientales, Universidad Autónoma del Carmen, C. 56 núm, 4 Esquina Avenida Concordia, Colonia Benito Juárez CP 24180, Ciudad del Carmen, Campeche, Mexico Universidad Autónoma del Carmen Campeche Mexico

**Keywords:** Fabaceae, Ingeae, Ingoid clade, mimosoid, New World, phylogenetic systematic, taxonomy

## Abstract

We present the most complete molecular phylogeny to date of the Pithecellobium clade of subfamily Caesalpinioideae. This neotropical group was informally recognised (as the Pithecellobium alliance) at the end of the 20^th^ century by Barneby and Grimes (1996) and includes five genera and 33 species distributed from the southern United States and Caribbean Islands to north-eastern South America. Our aims were to further test the monophyly of the group and its genera and to identify sister group relationships within and amongst the genera. A phylogenetic analysis of nuclear ribosomal DNA sequences (ITS and ETS) was performed. The results provide further support for the monophyly of the Pithecellobium clade. The genera *Ebenopsis*, *Pithecellobium* and *Sphinga* were strongly supported as monophyletic. *Havardia* and *Painteria* were found to be non-monophyletic, prompting their re-circumscriptions and the description of two new genera: *Gretheria* and *Ricoa*. New combinations are made for the three species transferred to the new genera.

## Introduction

In their seminal monographic treatment of the American synandrous mimosoid legumes, Barneby and Grimes (1996, 1997) significantly altered generic circumscriptions within Leguminosae tribe Ingeae (subfamily Caesalpinioideae; see LPWG 2017; Koenen et al. 2020), which, as traditionally defined, is clearly non-monophyletic, with part of tribe Acacieae nested within it (Luckow et al. 2003; Miller et al. 2003; Brown et al. 2008; LPWG 2017; Koenen et al. 2020). They also presented preliminary hypotheses on phylogenetic relationships amongst and within the treated genera. Most of the American species of the tribe were assigned to five informal alliances, each named for its most prominent genus (i.e. *Abarema* Pittier, *Chloroleucon* (Benth.) Britton & Rose, *Inga* Mill., *Pithecellobium* Mart. and *Samanea* (Benth.) Merr., respectively). Of these, only the so-called Pithecellobium alliance (henceforth called the Pithecellobium clade) has been consistently supported as monophyletic in subsequent molecular phylogenies (e.g. Brown et al. 2008; de Souza et al. 2013; Iganci et al. 2015; LPWG 2017; Koenen et al. 2020; Soares et al. 2021), albeit with limited taxonomic sampling. In a recent large-scale phylogenetic study of the mimosoid legumes by Koenen et al. (2020), the Pithecellobium clade was resolved within the so-called “ingoid clade”, where it belongs to a large clade comprising the majority of the genera formerly placed in tribe Ingeae plus *Acacia* (sensu Orchard and Maslin 2003).

The Pithecellobium clade consists of five genera: *Pithecellobium*, with 19 species, is the largest genus, followed by *Havardia* Small, with five species and *Ebenopsis* Britton & Rose, *Painteria* Britton & Rose and *Sphinga* Barneby & J.W. Grimes, each with three species. It is restricted to the tropics and subtropics of the New World, with species distributed from the southern United States and the Caribbean Islands to Peru and north-eastern Brazil. Its centre of species diversity lies in Mexico, which harbours all five genera and 18 species. The Antilles and South America each harbour eight species. Habitats include subtropical and tropical deciduous and semi-deciduous forests, thorny scrub, chaparral, desert grasslands and other xeromorphic vegetation, as well as coastal scrub and swamp forests, including mangroves. Amongst the American synandrous mimosoids, the clade is defined morphologically by sympodial growth, proleptic, dimorphic branches forming vegetative and/or reproductive short-shoots; spinescent stipules; buds protected by the adaxial side of the petiole; coeval or late-suppressed leaves; inflorescences with monomorphic flowers; and colporate non-equatorial pollen apertures (Barneby and Grimes 1996).

The five genera differ from each other most obviously in pod and seed characters (Barneby and Grimes 1996). In *Pithecellobium*, the pods are oblong to linear and recurved or coiled; following dehiscence, the seeds are suspended from the often-twisting valves by the funicle and the distal end of the funicle forms a red, pink or white fleshy aril cupping the lower third to half of the seed. The other four genera have non-arillate seeds. In *Ebenopsis*, the fruit is subterete, woody and internally septate between the overgrown (obese) seeds. In *Havardia* and *Sphinga*, the fruit is more strongly compressed and chartaceous to coriaceous, with the cavity continuous, the seeds not overgrown and the funicle sigmoid or contorted. In *Painteria*, the fruit is like in *Ebenopsis*, woody and subterete, but it is not internally septate between the plump lentiform seeds and the funicle is straight or sinuous. Another notable generic character is the presence of flask-shaped mature flower buds in *Sphinga* (vs. obovoid-pyriform in other genera).

**Figure 1.**
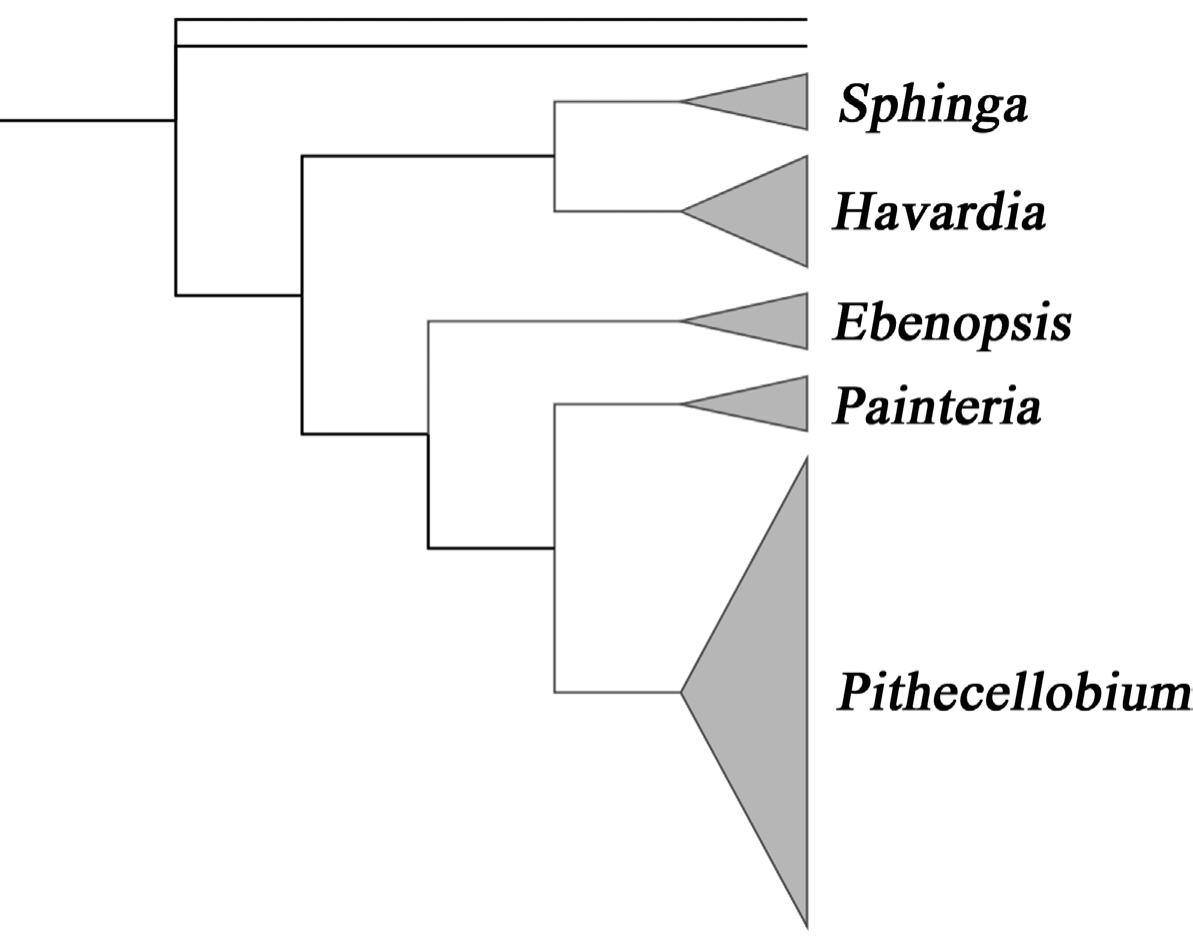
Summary cladogram based on maximum parsimony analyses of 29 morphological characters of the Pithecellobium clade (one of two trees resulting, the second involves differences in the internal topology of *Painteria*) from Barneby and Grimes (1996). Five genera were then recognised: Ebenopsis Britton & Rose (3 species), *Havardia* Small (5), *Painteria* Britton & Rose (3), *Pithecellobium* Mart. (19) and *Sphinga* Barneby & J. W. Grimes (3).

Barneby and Grimes (1996, 1997) undertook morphologically based phylogenetic analyses that included all taxa of the Pithecellobium clade. In their analyses, all five genera were recovered as monophyletic. *Sphinga* and *Havardia* formed a clade, which was, in turn, placed as sister to a clade containing the remaining three genera, within which *Painteria* and *Pithecellobium* were resolved as sister genera (Fig. 1). Hypothetical relationships (and morphological character state transformations) amongst and within the five genera were depicted in their fig. 12 of the first volume of their monograph (Barneby and Grimes 1996) and Fig. 1 of the second volume (Barneby and Grimes 1997).

To date, most molecular phylogenetic analyses that included species of the Pithecellobium clade have sampled single species of *Ebenopsis*, *Havardia*, *Pithecellobium* and *Sphinga* and no species of *Painteria* (e.g. Brown et al. 2008; de Sousa et al. 2013; Iganci et al. 2015; Koenen et al. 2020; Soares et al. 2021). Although each of these studies supported the monophyly of the group, with respect to relationships amongst the sampled genera, the analyses yielded mostly unresolved or poorly supported topologies (i.e. < 70% bootstrap support in parsimony analyses and/or < 0.95 posterior probabilities in Bayesian analyses). Three of these studies (Iganci et al. 2015; Koenen et al. 2020; Soares et al. 2021) strongly supported *Havardia* as the sister group of *Pithecellobium*.

Two molecular phylogenetic studies had substantially greater sampling of species of the Pithecellobium clade. The first, published as an electronic supplement to LPWG (2017), comprised an analysis of chloroplast *matK* sequences from a large and phylogenetically broad sample of species of Leguminosae (LPWG 2017), amongst which were two species of *Ebenopsis*, three of *Havardia*, one of *Painteria*, seven of *Pithecellobium* and two of *Sphinga*. Although the fine-scale details of the phylogenetic results were not discussed in the article, the analysis also recovered the Pithecellobium clade as monophyletic and resolved three well-supported clades, respectively grouping the two sampled species of *Sphinga*, the three sampled species of *Havardia* and six of the sampled species of *Pithecellobium*; the seventh *Pithecellobium* species, *P.keyense* was placed in an unresolved position and relationships amongst the genera were also unresolved.

The second study (Ringelberg et al. 2022, published in this special issue) is a phylogenomic analysis of subfamily Caesalpinioideae that produced an ASTRAL species tree, based on 821 single– or low-copy nuclear gene trees. It sampled all five genera and 11 species of the Pithecellobium clade, including the previously unsampled *Havardiacampylacantha* and *Painteriaelachistophylla*. The topology obtained is better resolved than that of LPWG (2017). It supports the monophyly of *Pithecellobium*, while rendering both *Havardia* and *Painteria* non-monophyletic.

Nevertheless, over half of the species of the Pithecellobium clade remain unsampled in these phylogenetic studies and, thus, knowledge of relationships within the group is still incomplete. Filling the sampling gaps is needed to establish a more robust phylogenetic framework for revising the classification of the group and, ultimately, for reconstructing its evolutionary history.

Here, we present the most comprehensively sampled molecular phylogenetic study of the Pithecellobium clade to date, based on analysis of sequences from nuclear ribosomal DNA regions. The following questions are addressed: 1) Are the Pithecellobium clade and its constituent genera monophyletic? 2) Does analysis of molecular data support the relationships amongst and within the genera recovered by previous analysis of morphology (Barneby and Grimes 1996, 1997)?

## Material and methods

### Taxon sampling and molecular markers

The ingroup sample included multiple representative species of all five genera of the Pithecellobium clade, for a total of 20 of the 33 species (61%) of the group (Table 1). Species were sampled from across the geographical range of the Pithecellobium clade. The number of species sampled per genus relative to total diversity was as follows: *Ebenopsis* (2/3), *Havardia* (5/5), *Painteria* (3/3) *Pithecellobium* (8/18) and *Sphinga* (2/3). Although less than half of the species of *Pithecellobium* were sampled, these included species of the three most basal lineages of the genus, as identified in the morphological analysis of Barneby and Grimes (1997). We were unable to sample the endemic Antillean species of *Pithecellobium* (e.g. *P.circinale* (L.) Benth., *P.cynodonticum* Barneby & J.W. Grimes and *P.histrix* (A. Rich.) Benth.), as well as some species from Central America (e.g. *P.furcatum* Benth. and *P.peckii* Blake). Based on the results of previous phylogenetic studies, the outgroup was composed of representative species of nine more distantly related mimosoid genera (Table 1; Brown et al. 2008; Kyalangalilwa et al. 2013; Iganci et al. 2015; Koenen et al. 2020). *Vachelliafarnesiana* (L.) Wight & Arn was used to root the analyses.

**Table 1.** Voucher information and GenBank accession numbers for the DNA sequences used in the present study.

	ETS	ITS
**outgroup**		
*Calliandraeriophylla*	MN755770.1	-
*Calliandrahaematocephala*	MN755769.1	JX870694.1
*Cojobaarborea*	MW849552.1	JX870758.1
*Cojobagraciliflora*	MW849557.1	MZ015531.1
*Faidherbiaalbida*	EF638163.1	JF270778.1
*Hesperalbiziaoccidentalis*	MN755774.1	MW699959.1
*Lysilomadivaricatum*	MN755783	MN755826
*Lysilomalatisiliquum*	MN755785	MN755827
*Mariosousadolichostachya*	EF638084.1	EF638199.1
*Vachelliafarnesiana*	EF638128.1	EF638219.1
*Zapotecaformosa*	MN755771	AY125854.1
*Zapotecatehuana*	MZ327390*A. Campos 4108* (MEXU)	OM634641*A. Campos 4108* (MEXU)
**Ingroup**		
*Ebenopsisconfinis*	MZ327411*A.L. Reina 696* (FCME)	KF921650.1
*Ebenopsisebano*	MZ327410*W. Torres et al. 84* (CICY)	EF638101.1
*Havardiaalbicans*	MZ327403*Duno 1945* (CICY)	OM634648*Duno 1945* (CICY)
*Havardiacampylacantha* (***Gretheriacampylacantha***)	MZ327405*E. Soto Núñez et al. 8036* (FCME)	OM634650*E. Soto Núñez et al. 8036* (FCME)
*Havardiamexicana*	MZ327397*T. R. van Devender 2005-1085* (MEXU)	JX870762.1
*Havardiapallens*	KF921656.1	EF638194.1
*Havardiasonorae* (***Gretheriasonorae***)	MZ327404*A. Flores 4875* (FCME)	OM634649*A. Flores 4875* (FCME)
*Painteriaelachistophylla*	MZ327409*García y Lorence 708* (FCME)	-
*Painterialeptophylla* (***Ricoaleptophylla***)	MZ327407*R. Cruz Durán 224* (MEXU)	OM634651*R. Cruz Durán 224* (MEXU)
*Painterialeptophylla 2* (***Ricoaleptophylla***)	MZ327406*J. Calónico 3751* (FCME)	*C.E. Hughes 1539* (FCME)
*Painteriarevoluta*	MZ327408*E. López 1107* (CICY)	-
*Pithecellobiumdiversifolium*	MZ327399*A. Laurenio 71* (MO)	JX870768.1
*Pithecellobiumexcelsum*	MZ327400*Tropical house Bot. Garden Aarhus* 2013	EF638208.1
*Pithecellobiumdulce*	OM674458*E. López* 1146 (CICY)	MZ015540.1
*Pithecellobiumkeyense*	MZ327394*Duno et al. 2216* (CICY)	OM634645*Duno et al. 2216* (CICY)
*Pithecellobiumlanceolatum*	MZ327398*E. Endañú 1310* (CICY)	-
*Pithecellobiumwinzerlingii*	MZ327393*Duno 2434* (CICY)	OM634644*Duno 2434* (CICY)
*Pithecellobiumoblongum*	MZ327396*I. Coronado & R.M. Rueda 5064* (MO)	OM634647*I. Coronado & R.M. Rueda 5064* (MO)
*Pithecellobiumunguis-cati*	MZ327395*H. M. Burdet & M. Burdet 02* (MO)	OM634646*H. M. Burdet & M. Burdet 02* (MO)
*Sphingaacatlensis*	MZ327391*E. López 1004* (CICY)	OM634642*E. López & E. Endañu 1020* (CICY)
*Sphingaplatyloba*	MZ327392*Duno et al. 2471* (CICY)	OM634643*Duno et al. 2471* (CICY)

### DNA sequences were gathered from two nuclear ribosomal regions, ETS and ITS

Thirty-two sequences were newly generated for this study, while 31 sequences were obtained from GenBank (www.ncbi.nlm.nih.gov/genbank); most of the latter were generated for the studies of Miller and Bayer (2001), Miller et al. (2003), Brown et al. (2008) and Duno de Stefano et al. (2021). The ETS dataset consisted of 34 sequences (21 new) from 33 species; the ITS dataset consisted of 29 (11 new) sequences from 30 species. GenBank accession numbers for all sequences are given in Table 1.

### DNA extraction, amplification and sequencing

Total genomic DNA was extracted from fresh leaves collected from the living collection in the Roger Orellana Regional Botanical Garden of the Centro de Investigación Científica de Yucatán, A. C., from leaflet tissue collected in the field and dried with silica gel or from herbarium specimens deposited in the following Herbaria: CICY, FCME, MA, MEXU, MO, UCOL and ZEA (acronyms as in Thiers (2020[and onwards]). Total DNA (from fresh or herbarium material) was obtained with DNeasy Plant Mini Kits (QIAGEN Inc., Valencia, California), following the manufacturer’s specifications. Concentration and relative quality of DNA was evaluated using the protocol in Duno de Stefano et al. (2021).

Amplifications were performed in an Applied Biosytems Veriti 96 Well Thermal Cycler (Applied Biosystems, Foster City, USA). Volumes of reagents in PCR reactions (all reactions were brought to final volume by adding ultrapure water) and cycling conditions were as follows for the two DNA regions: 1) ETS: 30 μl of mix containing 3 μl 10X Buffer, 2.5 μl MgCl_2_, 0.6 μl (~ 10 ng) each of primers, 4 μl Q solution, 1 μl 1.25 mM l-1 dNTP, 0.2 μl (1 U) TAQ polymerase, 2 μl (~ 10 ng) DNA; 94 °C for 3 min + 30 cycles (94 °C for 1 min + 60.5 °C for 1 min + 72 °C for 2 min) + 72 °C for 7 min; primers were 18S-IGS and 26S-IGS (Baldwin and Markos 1998) 2) ITS: 25 µl mix containing 2.5 µl 10X Buffer, 2.5 μl MgCl_2_, 0.6 μl (~ 10 ng) each of primers, 4 μl Q solution, 1 μl 1.25 mM l-1 dNTP, 0.2 μl (1 U) TAQ polymerase, 2 μl (~ 10 ng) DNA; 94 °C for 3 min + 30 cycles (94 °C for 1 min + 60.5 °C for 1 min + 72 °C for 2 min) + 72 °C for 7 min; primers were Ac 12F and Ac 1290R from Miller and Bayer (2001). PCR products (and the primers used for amplifications) were sent to Macrogen Korea, Seoul, South Korea for sequencing.

### Sequence assembly, alignment and molecular phylogenetic analyses

Assembly and editing of sequences were carried out in BioEdit v.7.0.9 (Hall 1999). Each of the two partitions was aligned independently in the online version of MAFFT (Katoh et al. 2002, 2017; https://mafft.cbrc.jp/alignment/server/) using the default settings (gap opening penalty = 1.53 and offset value = 0.00). Following exploratory phylogenetic analyses employing maximum parsimony, the concatenated alignment matrix was analysed using Bayesian Inference as implemented in MrBayes v. 3.2.7 (Huelsenbeck and Ronquist 2001; Ronquist and Huelsenbeck 2003), with each partition (ETS and ITS) treated as independent and associated with its own nucleotide substitution model. The best fitting model for each partition was selected in jModelTest v. 2.1.7 (Darriba et al. 2012), based on the Akaike Information Criterion (AIC). The following substitution models were selected: ETS = TIM3+I+G and ITS = GTR+I+G. Bayesian analyses consisted of two independent Metropolis-coupled Markov Chain Monte Carlo (MCMC) runs, each starting from a randomly chosen tree and run for five million generations, with one tree sampled every 2000 generations. Twenty percent of the sampled trees were discarded as burn-in after evaluation of the output parameters generated by the Bayesian analysis in Tracer v.1.6 (Rambaut et al. 2014). The remaining sampled trees were summarised in a 50% majority-rule consensus tree, with clade posterior probabilities (PP, i.e. the proportion of trees containing particular clades) used to measure clade support.

Posterior Probabilities of < 0.95 were considered weakly supported, whereas PP of 0.95–1.0 were deemed to be well supported. The convergence of MCMC runs was assessed with Tracer v. 1.7.1 (Rambaut et al. 2014) verifying that the effective sample size (ESS) for all parameters was > 200 (Nascimento et al. 2017).

## Results

The Bayesian analysis of ETS and ITS strongly supported the monophyly of the Pithecellobium clade (clade A in Fig. 3, PP = 1.0) and yielded a largely resolved topology within the group, with most nodes being well supported (Fig. 3). The genera *Ebenopsis*, *Pithecellobium* and *Sphinga* were each recovered as monophyletic with robust support values (clades L, G and F, respectively, PP = 0.98–1.0), but *Havardia* and *Painteria* were both resolved as non-monophyletic. Immediately above the base of the Pithecellobium clade, a marginally supported node (clade C, PP = 0.94) grouped a clade (E; PP = 1.0) comprised of three species of *Havardia* (*H.albicans*, *H.mexicana* and *H.pallens*) with *Pithecellobium* and *Sphinga*; the latter two genera were, in turn, well-supported as sister taxa (clade D; PP = 0.98). Within *Pithecellobium*, a well-supported clade (PP = 0.98), comprising *P.unguis-cati* and *P.oblongum*, was placed as the sister group to a poorly supported clade (PP = 0.86) containing the other sampled species of the genus. Two members of PithecellobiumsectionSpicata, *P.winzerlingii* and *P.lanceolatum*, were placed sister to each other (PP = 1.0) in this last clade. Another well-supported clade grouped the species *P.keyense* and *P.dulce* (as sister taxa) with *P.diversifolium* and *P.excelsum* (as sister taxa).

The other half of the basal dichotomy in the Pithecellobium clade was strongly supported (clade C, PP = 1.0) and grouped a clade (H, P = 1.0) containing two other species of *Havardia* (*H.campylacantha* and *H.sonorae*) with *Painteria* and *Ebenopsis*. Two of the three species of *Painteria* (*P.elachistophylla* and *P.revoluta*) formed a strongly-supported clade (K, PP = 1.0) that was placed as the sister group (clade J, P = 0.1) to *Ebenopsis*; the exclusion of *Painterialeptophylla* from this clade rendered *Painteria* paraphyletic.

## Discussion

### Comparison with previous studies

Our study shows both agreement and disagreement with well-supported results (PP ≥ 0.95 and/or likelihood or parsimony bootstrap ≥ 80%) of previous (Brown et al. 2008; Iganci et al. 2015; LPWG 2017; Koenen et al. 2020; Soares et al. 2021) and concurrent (Ringelberg et al. 2022) molecular phylogenetic studies. Our study is in agreement with all of these studies in strongly supporting the monophyly of the Pithecellobium clade. It also agrees with LPWG (2017) in supporting the monophyly of *Sphinga* and in the recovery of a clade grouping *Havardiaalbicans*, *H.mexicana* and *H.pallens*. With Ringelberg et al. (2022), which unlike the other studies sampled the key taxa *Havardiacampylacantha* and *Painteriaelachistophylla*, our study agrees in demonstrating the non-monophyly of both *Havardia* and *Painteria*, the latter due to the nesting of *Ebenopsis* within it and also agrees in supporting the monophyly of *Pithecellobium*.

Our results conflict with those of several studies by placing *Pithecellobium* as the sister taxon to *Sphinga* (Fig. 3). For example, the study of Iganci et al. (2015), although it sampled only single species of each genus (and none of *Painteria*), placed *Havardiapallens* sister to *Pithecellobiumdulce* (PP = 0.99), to the exclusion of *Sphingaacatlensis*. The same result, but even more strongly supported (100% likelihood bootstrap), was obtained by Koenen et al. (2020), while Ringelberg et al. (2022) had *H.pallens* sister to *Pithecellobium*, but with more species sampled for the later genus. Interestingly, Koenen et al. (2020) recovered a clade within which *Ebenopsisconfinis* was sister to *H.pallens* plus *P.dulce* (likelihood = 0.93, PP = 0.81), a result not recovered by any other analysis. The resolution of relationships within *Pithecellobium* is also somewhat conflicting between our study and those of LPWG (2017) and Ringelberg et al. (2022), although the overlap in sampling of species of the genus was limited between studies. For example, LPWG (2017) placed *P.keyense* in an unresolved position outside a clade comprising the other sampled species of the genus and Ringelberg et al. (2022) placed it sister to that clade, while in our phylogeny, *P.keyense* occupies a more nested position within *Pithecellobium*.

The causes of such conflict are unknown. Unlinked DNA regions used in the different studies may reflect different evolutionary histories, each with the potential to be differently impacted by evolutionary phenomena that cause phylogenetic conflict, such as gene duplication, hybridization and incomplete lineage sorting (Schrempf and Szöllősi 2020). Conversely, conflict may be attributable to statistical error, resulting from large differences in taxonomic sampling, the choice of sequence alignment criteria and/or the phylogenetic methods used, some of which may be more or less prone to phenomena, such as long branch attraction (Bergsten 2005). With respect to the present comparisons, we suspect that the latter is more prevalent since the other molecular phylogenetic studies of the Pithecellobium clade have less taxonomic sampling than our study. Moreover, we surmise that, in cases of conflict, our results are more compatible with previous taxonomic hypotheses, based on morphology.

Indeed, our study exhibits considerable agreement with the phylogenetic analysis of morphological data in Barneby and Grimes (1996, 1997), especially if the comparison is restricted to the clades from the morphological analyses that were supported by three or more putative morphological synapomorphies. For example, the monophyly of the alliance and the genera *Ebenopsis*, *Pithecellobium* and *Sphinga*, which were each strongly supported in our study, were also each supported by four to seven morphological synapomorphies in the analysis of Barneby and Grimes (1996, their fig. 12). Conversely, the two genera that were resolved as non-monophyletic in our phylogeny in conflict with Barneby and Grimes (1996), *Painteria* and *Havardia*, were respectively supported by only one and two putative morphological synapomorphies in the latter study. Within Pithecellobium, internal nodes supported by one or two morphological characters in Barneby and Grimes (1997, their Fig. 1) were not recovered by our analysis, whereas a node supported by seven morphological characters was resolved in our phylogeny (but with fewer sampled taxa) by the grouping of *P.lanceolatum* and *P.winzerlingii*. Even some clades supported by only one or two morphological characters in Barneby and Grimes (1996), such as a clade grouping *Havardiasonorae* and *H.campylacantha* and another clade grouping *H.albicans*, *H.mexicana* and *H.pallens*, were recovered in our analysis. However, one significant area of conflict involved the strongly supported sister relationship (PP = 1.0) between *Pithecellobium* and *Sphinga* in our study. In contrast, *Painteria* was resolved as the sister genus to *Pithecellobium* in Barneby and Grimes (1996), with four morphological characters supporting the result.

In cases of conflict between our molecular results and the morphological analyses of Barneby and Grimes (1996, 1997), we favour the former results for several reasons. First, although they mapped character state transitions on their phylogenies, Barneby and Grimes (1996) did not provide bootstrap values or other statistical measures of branch support in their phylogenies. Second, the morphological characters that were included in their phylogenetic studies included features, such as degree of pod compression, pod texture, pod curvature, valve reflection and coiling and degree development of an ovary stipe, which show continuous (or near continuous) variation when viewed across the entire Pithecellobium clade; thus, the division of the features into discrete states is subjective. Third, relative to molecular sequence data, suites of morphological characters may be more prone to homoplasy caused by shared selection pressures and/or developmental constraints (Scotland et al. 2003; Wiens 2004). Finally, relative to the phylogenies of Barneby and Grimes (1996), the well-supported phylogenetic results of our analyses show a greater degree of congruence with the results of previous molecular phylogenetic studies, including those based on analysis of other DNA regions (e.g. Brown et al. 2008; LPWG 2017; Koenen et al. 2020; Soares et al. 2021).

Other phylogenetic analysis in relation to the Pithecellobium clade sampled 15 species for *matK* (LPGW 2017) and 11 species for 997 nuclear genes (Ringelberg et al. 2022). They show different topologies for the Pithecellobium clade as well as the current topology (Fig. 3). The reasons for these differences could be due to the difference in the number of terminals, the number of nucleotides, alignment and analysis methods. Although the LPGW (2017) analysis has a few more terminals than Ringelberg et al. (2022), the latter has more key species (e.g. more taxa of *Havardia* and *Painteria*) and although this last study supports different generic relationships, it is congruent with the results presented here in demonstrating the non-monophyletic nature of *Havardia* and *Painteria*.

### Implications for the generic taxonomy of the *Pithecellobium* clade

Our results further substantiate that three of the five genera of the Pithecellobium clade, *Ebenopsis*, *Pithecellobium* and *Sphinga*, are monophyletic, whereas *Havardia* and *Painteria* are not. While there exist multiple taxonomic solutions that would yield a classification consisting of only monophyletic genera (see Backlund and Bremer 1998; Humphreys and Linder 2009), we strongly favour an option that preserves the first three genera. Beyond minimising nomenclatural changes, such a solution is desirable since the first three genera are each morphologically distinctive and easily diagnosed.

**Figure 2.**
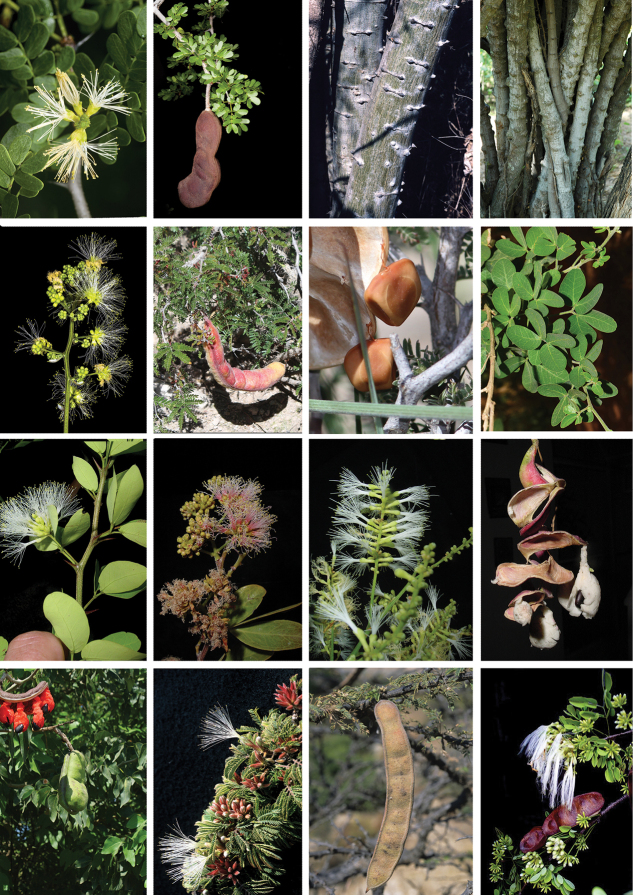
Morphological diversity of the genera of the Pithecellobium clade as circumscribed here. *Ebenopsisebano* (Berland.) Barneby & J.W. Grimes **A** inflorescence **B** pod. *Gretheriacampylacantha* (L. Rico & M. Sousa) Duno & Torke **C** bark. *Pithecellobiumexcelsum* (Kunth) Mart. **D** bark. *Havardiaalbicans* (Kunth) Britton & Rose **E** inflorescence. *Painteriaelachistophylla* (A. Gray ex S. Watson) Britton & Rose **F** pod **G** seed. *Pithecellobiumdulce* (Roxb.) Benth. **H** leaves. *Pithecellobiumwinzerlingii* Britton & Rose **I** inflorescence. *Pithecellobiumkeyense* Britton **J** inflorescence. *Pithecellobiumlanceolatum* (Humb. & Bonpl. ex Willd.) Benth. **K** inflorescence. *Pithecellobiumunguis-cati* (L.) Benth. **L** pod and seed. *Pithecellobiumlanceolatum* (Humb. & Bonpl. ex Willd.) Benth. **M** pod and seed. *Sphingaacatlensis* (Benth.) Barneby & J.W. Grimes **N** branch, leaves and inflorescences **O** pod. *Sphingaplatyloba* (Bertero ex DC.) Barneby & J.W. Grimes **P** leaves and inflorescence. Photos: **A, B, I–K, P** German Carnevali **C, D, N, O** Colin E. Hughes **E, L** Gustavo A. Romero **F, G** Pedro Najéra Quezada, https://www.naturalista.mx) **H** Peter Pedersen **M** Rodrigo Duno.

**Figure 3.**
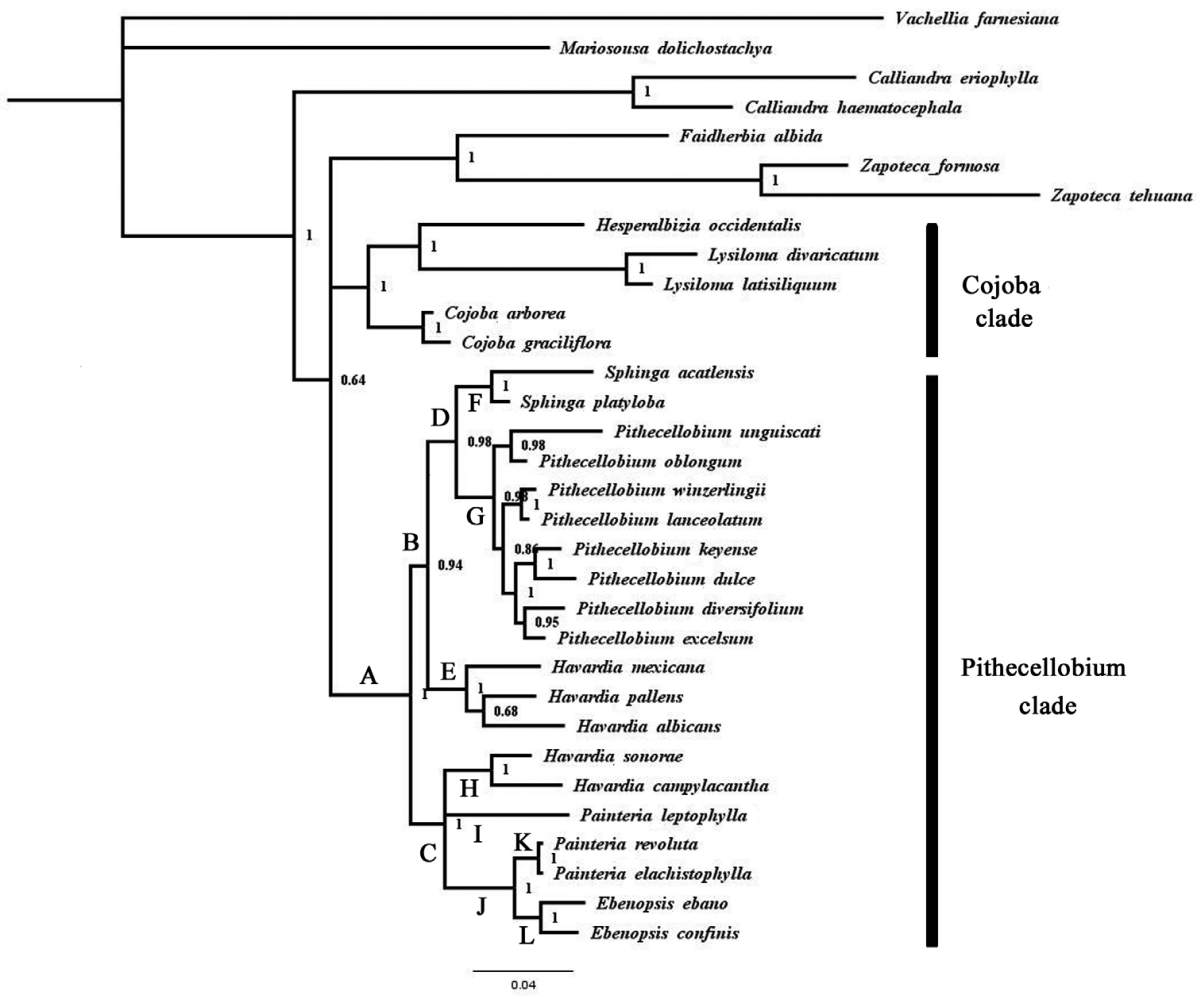
Results of majority rule consensus tree of the Bayesian analysis of the nuclear ribosomal ETS and ITS regions the clade Pithecellobium.

There are two other nomenclatural options, which, in our opinion, are less appropriate. The first one would be to include all members of clade C (Fig. 3) - *p.p.* (excluding the type), *Ebenopsis* and *Painteria* - in a single genus named *Ebenopsis*, which has nomenclatural priority. However, this would result is a heterogeneous genus with respect to fruit morphology (Figs 2b, f and n), habit and ecology. The second option would be to recognise members of clade H (Fig. 3) as a new genus (as we do here) to transfer *Painteriaelachistophylla* and *P.revoluta* to *Ebenopsis*. This new circumscription of *Ebenopsis* reduces morphological variation, but retains notable differences in habit and ecology. While *Ebenopsis*, although less expansive than in the first option, nevertheless encompasses trees growing in lowlands, *Painteria* comprises shrubs growing in highlands of up 2600 m elevation.

As for *Havardia* and *Painteria*, the preferred option retains these generic names for the clades that include the generic type species, *H.pallens* and *P.revoluta*, respectively. It thus requires the erection of two new genera: one for the clade comprised of *H.sonorae* and *H.campylacantha*, another for the species *P.leptophylla*. These four genera (the re-defined *Havardias.s.* and *Painterias.s.* and the proposed new genera) are individually diagnoseable by morphology.

*Havardia*, as here circumscribed, can be diagnosed within the complex for having both sylleptical and proleptical shoots, inflorescences arising from long shoots, leaves with one pair of pinnae and flowers with recurved corolla lobes. The new genus that we name *Gretheria* (containing *H.sonorae* and *H.campylacantha*) also has both sylleptical and proleptical shoots and inflorescences arising from long shoots, but the pinnae are distally accrescent and the corolla lobes are erect-ascending.

*Painteria* as redefined here, could be diagnosed by the combination of the valves of the pod elastically reflexed with age, the leaves with just one pair of pinnae and the corolla lobes erect-ascending, whereas the new monotypic genus *Ricoa* (comprising *P.leptophylla*), while also having the valves elastically reflexed with age, has leaves with the pinnae decrescent at each end and the corolla lobes recurved.

In the taxonomic treatment that follows, we provide descriptions (drawing heavily from data in Barneby and Grimes 1996) for the two new genera and make the necessary new combinations for the three species contained within them. Table 2 summarises the diagnostic characters of the seven recognized genera of the Pithecellobium clade.

**Table 2.** Morphological comparison between the genera of the Pithecellobium clade as circumscribed here (modified from Barneby and Grimes 1996, 1997).

	*Ebenopsis*	*Gretheria*	*Havardia*	*Painteria*	*Pithecellobium*	*Ricoa*	*Sphinga*
Habit	Tree or shrub	Shrub or tree	Tree	Shrub	Tree or shrub	Shrub	Shrub or tree
Leaflets, size	Microphyllous	Microphyllous	Microphyllous	Microphyllous	Micro- to Macrophyllous	Microphyllous	Macrophyllous
Branching, pattern	Proleptically	Sylleptically and proleptically	Sylleptically and proleptically	Proleptically	Proleptically	Proleptically	Proleptically
Extrafloral gland, position	Interpinal	Near midpetiole,	Interpinal but the first below proximal pinna-pair or near midpetiole	Between proximal pinna-pair, rarely between 2 pairs, not on petiole proper	Interpinal	between the first pinnae pair	Near midpetiole, rarely at tip of petiole
Leaflet, venation	Palmate	Pinnate	Pinnate, weakly pinnate, simple, weakly palmate-pinnate	Palmate or simple weakly developed superficially	Pinnate and usually also reticulate	Weakly developed, nearly simple or 1-branched	Pinnate or subpalmate
Inflorescence	Capitula or shortly spiciform	Capituliform racemes	Capitula or spiciform	Capitula or shortly spiciform	Capitula or spikes	Capitula	Capitula
Flower, anthesis	Diurnal	Vespertine	Diurnal	Diurnal	Diurnal	Vespertine	Vespertine
Corolla, lobes	Erect	Erect	Recurved	Erect	Erect	Recurved	Erect
Disc (ovary)	Absent	Simple callosities or 5-lobed	Absent, obsolete callosities, rarely lobed disc	Callosities obsolete or absent	Callosities obsolete, rarely developed into a lobed disc	Callosties developed or sometimes subobsolete (staminate flowers)	Developed, clasping the stipe
Funiculus, shape	Straight (not sigmoid)	dilated, sigmoid	Sigmoid	Straight or sinuous (not sigmoid)	Spongy arilliform	Straight or sinuous (nor sigmoid)	Contorted or sigmoid
Fruit, shape	massive, compressed, sausage-like	Oblong plano-compressed	Oblong or broad-linear straight, plano-compressed	Compressed but turgid, retrofalcate, falcately or subcircinnately broad-linear	oblong or linear in profile, backwardly recurved or coiled and sometimes also twisted	Falcately or subcircinnately broad-linear	Broad-linear plano-compressed
Fruit, consistence	Woody	Stiff	Chartaceous or thinly coriaceous	Leathery	Leathery or woody	Stiffly leathery	Papery
Fruit, septation	Yes	No	No	No	No or incipient	No	No
Seed	Plumply obese	disciform to orbicular	Lentiform, orbicular or oblong-elliptic	Plumby lentiform	plumply	Compressed but plumb	Lentiform

## Taxonomy

### Key to the genera of the *Pithecellobium* clade (modified from Barneby and Grimes 1996)

**Table:** 

1	Trees or shrubs, generally sarmentous; petiolar gland in the mid-petiole; flower bud flask-shaped, flowers opening at night; androecium up to 9 cm long	***Sphinga***
–	Trees or shrubs, erect never sarmentous; petiolar gland at the point of origin of the first (or only) pair of pinnae or in the mid-petiole; flower bud ovoid-pyriform, flowers opening during day; androecium usually less than 3 cm long, very rarely up to 7 cm long	**2**
2	Petiolar gland below the first pair of pinnae; fruits flattened, papery and straight	**3**
–	Petiolar gland between the first pair of pinnae; fruits never flattened, never papery and occasionally straight (then woody or sub-woody)	**4**
3	Flowers with calyx 1.0–2.0 mm long, teeth 0.25–1.5 mm long, shallowly campanulate; corolla lobes recurved in anthesis; ovary disc absent	***Havardia***
–	Flowers with calyx 2.8–3.4 mm long, teeth 0.3–0.8 mm long, deeply campanulate; corolla lobes erect in anthesis; ovary disc present (sometimes poorly developed)	***Gretheria***
4	Leaves with one pair of pinnae and leaflets one pair per pinna or leaves with more than two pairs of pinnae and leaflets two to many pairs per pinna; seeds with fleshy, often brightly coloured arils	***Pithecellobium***
–	Leaves with two or more pairs of pinnae, never one, leaflets 2–30 pairs per pinna; seeds without aril	**5**
5	Pod cylindrical, woody, straight or slightly curved, deeply internally septate; seeds globose; growing in lowlands of Mexico and the United States (Texas)	***Ebenopsis***
–	Pod flattened or slightly subterete, sub-woody and curved, without internal septa; seeds lentiform; growing in highlands of Mexico	**6**
6	Leaves with one or two pairs of pinnae; leaflets 3 to 10 per pinna, rarely 12; blades suborbicular, broadly oblong or elliptic (then revolute); corolla lobes ascending	***Painteria***
–	Leaves with 3–7(9) pairs of pinnae; leaflets 10 to 25 per pinna; blades narrowly oblong, linear-oblong or lanceolate; corolla lobes recurved	***Ricoa***

### Gretheria

Taxon classificationPlantaeFabalesFabaceae

Duno & Torke gen. nov.

AEBCCA5C-A036-55D2-B77C-F42870B7FB8E

urn:lsid:ipni.org:names:77303792-1

#### Diagnosis.

Similar to *Havardia* in arboreal or shrubby habit, vegetative branches arising both proleptically and sylleptically, leaves microphyllous, inflorescence arising on long shoots, pod flattened-compressed and seed plumply disciform to orbicular, but differing in the pinnae distally accrescent (vs. decrescent at each end in *Havardia*), the calyx longer and deeply campanulate (vs. shorter, and shallowly campanulate), and the corolla lobes erect-ascending at anthesis (vs. recurved).

#### Type.

*Gretheriasonorae* (S. Watson) Duno & Torke.

#### Description.

Xerophytic, microphyllous arborescent shrubs and small trees, 2–14 m tall, commonly armed with stout recurved, lignescent stipules on the trunk and at each node of long-shoots, indumented with minute whitish trichomes on new growth. ***Leaves*** bipinnate, with 1–6 (13) pairs of pinnae; leaflets 10–31 per pinna; principal leaf axis typically 2–15 cm long, with the petiole 2–24 mm long, bearing a nectary at or below the mid-point of the petiole, the nectary sessile, shallow-cupular, thick-rimmed or plane and dimpled, pinnae axes sometimes with similar but smaller nectaries between 1–2 (3) distal-most pinna pairs and a minute one at the tip of some pinna-rachises; leaflets opposite, the blade oblong-elliptic to linear-oblong, subcordate at base, obtuse or shortly apiculate at apex; pilosulous or glabrous and marginally ciliolate; venation pinnate, immersed above, prominulous beneath, the mid-rib slightly displaced, giving rise on each side to 2–5 weak secondary veins expiring submarginally or faintly brochidodromous. ***Inflorescence*** capituliform racemes arising from leaf axils of long shoots and coeval with or preceding the leaf and/or arising from brachyblasts; peduncles (1.3) 2 cm long; capitula 10–37-flowered, receptacle clavate, 1.5–2.5 mm diameter; bracts ovate, minute, less than 1 mm long, sessile, persistent into anthesis. ***Flowers*** sessile, homomorphic, the perianth 5-merous; calyx deeply campanulate, glabrous, teeth deltate-ovate, ciliolate and sometimes distally puberulent or strigose; corolla subcylindrical, lobes erect, white-silky strigose dorsally; androecium 40–52-merous, 9–13 mm long, tube 3.6–5 mm long, nectar disc simple callosities or 5-lobed, 0.2 – 0.35 mm tall; ovary subsessile, slenderly ellipsoid, stipe 0.1–0.25 mm long; style about as long as stamens, the stigma poriform. ***Pods*** 1–3 per capitulum, oblong in profile, contracted at base into a pseudostipe ± 5–14 mm long and abruptly so at apex into an erect cusp 1.5–8 mm long; body straight or almost straight, 6.5–13 × (1.3) 1.2–2.4 (2.6) cm, plano-compressed, the valves bluntly framed by longitudinally 3-ridged sutures ±1.5–2 mm wide, stiff, somewhat brittle, brownish-green, externally veinless, glabrous, red-granular or both granular and puberulent outside, the cavity continuous; funicle dilated, sigmoid. ***Seeds*** transverse, 8–13, plumply disciform to orbicular in outline, 9–12 × 7–10 mm, the pleurogram U-shaped.

#### Geographic distribution.

*Gretheria* comprises two species in United States (Texas), Mexico and Central America (Honduras and Nicaragua).

#### Habitat.

*Gretheria* grows in tropical deciduous dry forests, thorn scrubs and brush-woodlands, between sea level and 400 m elevation, occasionally ascending to 700 m.

#### Etymology.

The generic name honours Rosaura Grether González, an extraordinary and prolific Mexican botanist, with whom we had the pleasure of sharing her experience as a botanist and colleague. Her profound dedication and perseverant commitment to botanical research over decades has contributed importantly to our knowledge and understanding of Leguminosae, especially of the genus *Mimosa* L.

### Key to the species of *Gretheria* (modified from Barneby and Grimes 1996)

**Table:** 

1	Petiole 8–24 mm long; leaves with (5) 6–11 (13) pairs of pinnae, leaflets (17) 19–31 per pinna; leaflets linear or linear-oblong, 3.3–6.5 × 0.8–2 mm; capitula 12–37-flowered	***Gretheriacampylacantha***
–	Petiole 2–7.5 mm long; leaves with 1–5 pairs of pinnae, leaflets (10) 13–21 per pinna; leaflets narrowly oblong or oblong-elliptic, 2.2–5.5 × 0.8–1.3 mm; capitula 10–17-flowered	***Gretheriasonorae***

### 1.1. Gretheria campylacantha

Taxon classificationPlantaeFabalesFabaceae

(L. Rico & M. Sousa) Duno & Torke comb. nov.

5B2FE561-6EE3-5211-BEC1-DD79A1B658CB

urn:lsid:ipni.org:names:77303793-1

#### Basionym.

*Pithecellobiumcampylacanthum* L. Rico & M. Sousa (as “*campylacanthus*”), Ann. Missouri Bot. Gard. 73: 722–724. 1986[1987]. *Havardiacampylacantha* (L. Rico & M. Sousa) Barneby & J.M. Grimes (as “*campylacanthus*”), Mem. New York Bot. Gard. 74(1): 167. 1996.

#### Type.

México. Oaxaca, distrito de Tehuantepec, 7 km al O-NO de Tehuantepec, 17 March 1981, *M. Sousa et al. 11938* (holotype: MEXU! 410015; isotypes: BM, CAS accession 0004063 [image!], F accession 2064374 [image!], MEXU accessions 41011 [image!], 410013 [image!], 410016 [image!], MO accession 3481860 [!]).

#### Geographic distribution.

*Gretheriacampylacantha* occurs discontinuously in the Pacific lowlands of south-eastern Mexico (Guerrero, Michoacán and Oaxaca) and in the interior and Pacific lowlands of Central America (from Comayagua Department in Honduras to Boaco Department in Nicaragua).

#### Habitat.

It grows in tropical deciduous brush-woodlands, along intermittent streams, between sea level and 200 m elevation, occasionally ascending to 700 m.

### 1.2. Gretheria sonorae

Taxon classificationPlantaeFabalesFabaceae

(S. Watson) Duno & Torke comb. nov.

77D0E4DF-097D-5B4F-9982-402EE818DE27

urn:lsid:ipni.org:names:77303794-1

#### Basionym.

*Pithecellobiumsonorae* S. Watson, Proc. Amer. Acad. Arts 24: 49. 1889. *Havardiasonorae* (S. Watson) Britton & Rose, N. Amer. Fl. 23: 42. 1928.

#### Type.

México. Sonora, common at Guaymas 1887, *E. Palmer 58* (holotype: GH accession 00064044 [image!]; isotypes: K accession 000082458 [image!], NDG 46766 [image!], NY accessions 00329628, 00329629 [images!], UC accession 84451 [image!], US accessions 00918587, 00918589 [image!], YU accession 001418 [image!]).

#### Geographic distribution.

Coastal plain of Baja California Sur, Sonora and Sinaloa in Mexico.

#### Habitat.

Plains and foothills below 400 m in deciduous dry forest and thorn scrub and along washes in mesquite grassland along the coastal plain.

### Ricoa

Taxon classificationPlantaeFabalesFabaceae

Duno & Torke gen. nov.

D822D032-54A3-5F56-822E-45BE84650C9C

urn:lsid:ipni.org:names:77303795-1

#### Type.

*Ricoaleptophylla* (DC.) Duno & Torke.

#### Diagnosis.

Similar to *Painteria* in shrubby habit with pronounced growth dimorphism into long- and short-shoots, deciduous microphyllous leaves and recurved pods with the fruit valves coriaceous to lignescent and elastically reflexed with age, but differing in the leaves with 3–7(9) pairs of pinnae (vs. 1–2 in *Painteria*), the leaflets 10–25 per pinna (vs. 3–12), the floral bracts 0.8–2.1 mm long (vs. 0.4–0.7 mm), the flowers shortly pedicellate (vs. sessile) and the corolla lobes recurved (vs. erect-ascending).

#### Description.

Low xerophytic stiffly branched microphyllous shrubs 2–1.5 m tall, often growing in patches several metres in diameter, armed at each node of flexuous long shots with a pair of lignescent stipules, young growth indumented with minute whitish hairs. ***Stipules*** converted with straight to recurved spines with a thickened base, the spines 3–10 mm long. ***Leaves*** bipinnate with 3–7 (9) pairs of pinnae; leaflets 8–25 pairs per pinna, the primary leaf axis 0.5–5 cm long, with the petiole 2.5–10 (18) mm long and a subsessile circular nectary between the first pinnae pair (sometimes also between the second pair), nectaries absent on pinna-rachises; leaflets opposite, narrowly- or linear-oblong or lanceolate, semi-cordate at base, obtuse to weakly apiculate at apex, puberulous abaxially, marginally ciliate, the venation weakly developed, nearly simple or 1-branched, the mid-rib prominulous only dorsally, subcentric. ***Inflorescence*** of capitula arising from brachyblasts, peduncle 1–18 mm long; receptable clavate, 1.5–2.5 mm long, capitula globose, 1–1.5 cm in diameter, 16–35-flowered; bracts linear-oblanceolate or spatulate, 0.8–2.1 mm long, persistent into anthesis. ***Flowers*** externally homomorphic, but some functionally staminate, pedicellate, perianth 5-merous; pedicel 0.2–0.6 mm long; calyx campanulate, contracted at base, 1.3–3.2 mm long, minutely puberulent (or just on teeth), teeth ovate or deltate, 0.2–0.9 mm long; corolla reddish or greenish, tubular, 3.5–5 mm long, lobes ovate, recurving. 1.2–1.9 mm long, puberulous and densely fimbriolate on lobes; androecium 40–76-merous, 5–10.5 mm long, tube 2–4 mm long; ovary slenderly ellipsoid, compressed, glabrous, on a short stipe 1–1.4 mm long, style in bisexual flowers often longer and more robust than stamens. ***Pods*** l–2 (4) per capitulum, falcately or subcircinnately broadly linear in profile, attenuate into an erect cusp 2–6 mm long, the body 7–11.5 × 1.1–1.9 cm, 8–10-seeded, the valves stiffly leathery, at first plano-compressed, becoming turgid and low-convex (on both faces of pod) over each seed, densely grey puberulent, becoming glabrescent and dark castaneous, indistinctly venulose, the cavity continuous, dehiscence inert through both sutures; funicle straight or sinuous (but not sigmoid), seeds obliquely descending, 8–10, plumply lentiform, 7.5–11 × 3–4 mm, the testa smooth, hard, moderately lustrous, dark castaneous, the pleurogram incomplete.

#### Distribution.

*Ricoa* is found scattered over the Mexican Central Plane, in the States of Chihuahua, Coahuila, Durango, Guanajuato, Hidalgo, Jalisco, México, Michoacán, Oaxaca, Puebla, Querétaro, San Luis Potosí, Tlaxcala and Zacatecas.

#### Habitat.

It grows in grasslands, scrubs and at the lower edge of the pine-oak belt, on both basaltic and calcareous substrates, at 1600–2800 m elev. Plants flower between March and August.

#### Common names.

The common name is Huisache. This name is also given to *Vachelliafarnesiana* (L.) Wight & Arn. and other related species (Barneby and Grimes 1996). Other common names are charrasquillo, gatuña and tehuixtle (Calderón de Rzedowski 2007).

#### Etymology.

The generic name honours María Lourdes Rico, whose profound dedication and perseverant commitment to botanical research over decades has deeply enhanced knowledge and understanding of the Leguminosae, especially tribe Ingeae.

### 2.1. Ricoa leptophylla

Taxon classificationPlantaeFabalesFabaceae

(DC.) Duno & Torke comb. nov.

52C1DBD7-F029-5BCB-8615-49A3C4B458AD

urn:lsid:ipni.org:names:77303796-1

#### Basionym.

*Acacialeptophylla* DC., Cat. PI. Horti Monsp. 74. 1813. *Mimosaleptophilla* [sic] Cavanilles, Elench. PI. Horti Matr. 24. 1803, *nom. nud*. *Pithecellobiumleptophyllum* (DC.) Daveau, Bull. Soc. Bot. France 59: 635, t. XVI. 1912. *Painterialeptophylla* (DC.) Britton & Rose, N. Amer. Fl. 23: 36. 1928.

#### Type.

Mexico. verosimiliter in Hispanorum territorio Americano, *P. M. A. Broussonet s.n.* [870]. (holotype: M; isotypes: G-DEL [image!], photo, MO [image!]).

## Supplementary Material

XML Treatment for Gretheria

XML Treatment for Gretheria campylacantha

XML Treatment for Gretheria sonorae

XML Treatment for Ricoa

XML Treatment for Ricoa leptophylla
